# Genome-wide association study and Mendelian randomization analyses reveal insights into bladder cancer etiology

**DOI:** 10.1093/jncics/pkaf014

**Published:** 2025-02-03

**Authors:** Susanna C Larsson, Jie Chen, Xixian Ruan, Xue Li, Shuai Yuan

**Affiliations:** Medical Epidemiology, Department of Surgical Sciences, Uppsala University, Uppsala, Sweden; Unit of Cardiovascular and Nutritional Epidemiology, Institute of Environmental Medicine, Karolinska Institutet, Stockholm, Sweden; Department of Big Data in Health Science School of Public Health, Center of Clinical Big Data and Analytics of The Second Affiliated Hospital, Zhejiang University School of Medicine, Hangzhou, China; Department of Gastroenterology, The Third Xiangya Hospital, Central South University, Changsha, China; Department of Big Data in Health Science School of Public Health, Center of Clinical Big Data and Analytics of The Second Affiliated Hospital, Zhejiang University School of Medicine, Hangzhou, China; Unit of Cardiovascular and Nutritional Epidemiology, Institute of Environmental Medicine, Karolinska Institutet, Stockholm, Sweden

## Abstract

**Background:**

The causes of bladder cancer are not completely understood. Our objective was to identify blood proteins and modifiable causal risk factors for bladder cancer by combining genome-wide association study (GWAS) and Mendelian randomization (MR) analyses.

**Methods:**

We first performed a GWAS meta-analysis of 6984 bladder cancer case patients and 708 432 control individuals from 3 European databases. Next, we conducted 2-sample MR and colocalization analyses using data from the present GWAS and published GWAS meta-analyses on plasma proteins and modifiable factors.

**Results:**

Genome-wide association study meta-analysis uncovered 17 bladder cancer susceptibility loci, of which 3 loci were novel. Genes were enriched in pathways related to the metabolic and catabolic processes of xenobiotics and cellular detoxification. Proteome-wide MR analysis based on *cis*-acting genetic variants revealed that higher plasma levels of glutathione *S*-transferases were strongly associated with a reduced risk of bladder cancer. There is strong evidence of colocalization between GSTM1 and bladder cancer. Finally, multivariable MR analyses of suspected risk factors for bladder cancer revealed independent causal associations between smoking and adiposity, particularly abdominal obesity, and risk of bladder cancer.

**Conclusions:**

Findings from this large-scale GWAS and multivariable MR analyses highlight the key role of detoxification processes, particularly glutathione *S*-transferase 1, as well as smoking and abdominal obesity in bladder cancer etiology.

## Introduction

Bladder cancer is a common malignancy, with approximately half a million incident case patients diagnosed globally in 2019.^1^ The risk of bladder cancer increases with advancing age, and men and smokers are more often afflicted than women and nonsmokers.^1-3^ Approximately one-third of all bladder cancer case patients are attributable to smoking.^1^ Other potential causes of bladder cancer include certain chemicals such as benzene^4^ and aromatic amines,^2^^,^^5^ as well as drinking water containing arsenic.^6^ Observational studies have further reported a modestly increased risk of bladder cancer associated with elevated fasting glucose levels,^1^ type 2 diabetes,^7^^,^^8^ and excess leisure sitting time^9^ and a decreased risk of this malignancy among people who report moderate and vigorous physical activity.^9^^,^^10^ Evidence on the association of other potentially modifiable risk factors, such as adiposity,^11^ alcohol consumption,^12^^,^^13^ and coffee consumption,^14^^,^^15^ with bladder cancer risk is inconclusive. Given the observational design of most previous studies, the causality of the associations between modifiable factors and bladder cancer risk remains unclear.

Plasma proteomics is a technique that can uncover critical pathways for cancer development and identify therapeutic targets for cancer prevention. Proteins play key roles in many cellular processes and functions, such as detoxification of endogenous and environmental compounds, signaling and metabolic pathways, DNA repair, and apoptosis. However, the role of the plasma proteome in bladder carcinogenesis has not been fully elucidated.

This study aimed to identify potential therapeutic targets and lifestyle interventions for bladder cancer prevention by combining data from a genome-wide association study (GWAS) on bladder cancer and GWAS data on plasma proteins and modifiable factors with a suspected association with bladder cancer risk. Key findings from the GWAS meta-analysis are presented.

## Methods

### Study design and data sources

This study was initiated by a meta-analysis of imputed genotyped data for 6984 bladder cancer case patients and 708 432 control individuals from 3 European studies, including the UK Biobank,^16^ FinnGen,^17^ and Swedish Infrastructure for Medical Population-based Life-course and Environmental Research (SIMPLER) cohorts.^18^ We used individual genotype-level data for the UK Biobank and SIMPLER cohorts, and summary level data for FinnGen. Case patients were defined based on the International Classification of Diseases codes 188 for the eighth and ninth revisions and C67 for the 10th revision in all studies. Information on these studies, genotyping, imputation, and quality control has been provided in detail in previous articles^16-18^ and briefly in the Supplementary Material S1.

By leveraging data from this GWAS meta-analysis and previous GWAS meta-analyses of plasma proteins and potentially modifiable risk factors, we performed 2-sample univariable and multivariable Mendelian randomization (MR) analyses to isolate the causal proteins and independent modifiable risk factors for bladder cancer. Mendelian randomization is a tool that employs genetic variants as instrumental variables for exposure to improve causal inferences in observational data.

All studies included in the GWAS meta-analysis obtained informed consent from study participants and approval from an ethical review authority. The Swedish Ethical Review Authority approved the analysis conducted in this study.

### Statistical analysis

#### GWAS meta-analysis and enrichment analysis

We used METAL^19^ to meta-analyze data from 3 studies using a fixed-effects model and tested heterogeneity in association estimates across studies. Linkage disequilibrium (LD) score regression was conducted to quantify the genomic inflation factor in each study.^20^ Functional Mapping and Annotation of Genome-Wide Association Studies (FUMA)^21^ were used to discern independent genomic loci using the 1000 Genomes Phase III European dataset for LD computations. The thresholds were set at genome-wide significance (*P* < 5 × 10^−8^), physical distance at window above 500 kb, and LD at *r*^2^ = 0.6 and $r_{2}^{2}$ = 0.1. The lead single-nucleotide polymorphism (SNP) representing the locus was defined as the variant with the smallest *P*-value. We searched the National Human Genome Research Institute - the European Bioinformatics Institute Catalog until April 30, 2024, and a recent GWAS meta-analysis^22^ to identify previously reported bladder cancer susceptibility loci. To map SNPs onto genes, we used multivariate analysis of genomic annotation with the maximum window size (50 kb).^23^ We performed a transcriptome-wide association study (TWAS) using FUSION software^24^ based on data from the Cancer Genome Atlas Program, including 380 patients with bladder urothelial carcinoma. A total of 1677 genes were included in the TWAS. We then performed pathway enrichment using data from the Kyoto Encyclopedia of Genes and Genomes^25^ based on genes identified in TWAS after false discovery rate (FDR) multiple testing correction. Tests of statistical significance in all analyses were 2-sided.

#### MR analyses

We performed a proteome-wide MR analysis to identify the plasma proteins with a potential causal role in bladder cancer development. To reduce pleiotropy and other biases, we selected *cis*-acting (ie, SNPs proximal to the gene encoding the protein) protein quantitative trait loci (pQTLs) from 2 independent GWASs^26^^,^^27^ without sample overlap with the bladder cancer GWAS meta-analysis. For each protein, the index *cis*-pQTL with the strongest association with the protein was selected as the instrument. We used instruments obtained from the Diabetes Epidemiology: Collaborative analysis Of Diagnostic criteria in Europe (deCODE) (*n* = 35 559 participants, data collection 2000-2019)^26^ and Fenland (*n* = 10 708 participants, data collection 2005-2015)^27^ studies for the discovery and replication analysis, respectively. Plasma proteins were profiled using the SomaScan version 4 assay (SomaLogic) in both studies.

We conducted 2-sample MR analyses to examine the potential causal associations of suspected modifiable risk factors for bladder cancer,^1^^,^^7-15^ including 3 measures of adiposity (body mass index, waist-to-hip ratio, and visceral adiposity), type 2 diabetes, fasting glucose, fasting insulin, and lifestyle factors (ie, two smoking traits [smoking initiation and a lifetime smoking index], alcohol and coffee consumption, moderate to vigorous physical activity, and leisure screen time). Information on the GWAS meta-analyses of modifiable risk factors is provided in Table S1.

As instrumental variables for the modifiable factors, we selected SNPs associated with each exposure at a *P* below 5 × 10^−8^. For SNPs in LD (*r*^2^ > 0.01), only the SNP with the strongest association with exposure was retained. To measure the strength of the genetic instruments, we computed the *F*-statistic for each modifiable factor. The overall *F*-statistic was above 30 for all instruments. The SNPs employed as instrumental variables and the relevant summary statistics are provided in Table S2.

The Wald ratio method was used to obtain MR estimates for the protein-bladder cancer associations. For modifiable factors, we applied the multiplicative random-effects inverse variance weighted method as the main analytical approach. The weighted median,^28^ MR-Egger regression,^29^ and Mendelian Randomization Pleiotropy RESidual Sum and Outlier (MR-PRESSO)^30^ methods were used for sensitivity analyses. We evaluated the heterogeneity between estimates from individual instrumental variables using Cochran’s *Q* value and assessed pleiotropy using the MR-Egger intercept and MR-PRESSO global tests. To discern the independent association between modifiable risk factors, we performed multivariable MR analyses. We adjusted for multiple testing using the Benjamini-Hochberg FDR correction. Data harmonization and MR analyses were performed using the TwoSampleMR and MendelianRandomization packages in R. Tests of statistical significance in all MR analyses were 2-sided.

#### Colocalization analysis

As a supplement to *cis*-pQTL-based MR, we performed genetic Bayesian colocalization analysis to further eliminate potential confounding by LD. We included SNPs above or below 500 kb from the *cis*-pQTL. We tested 5 hypotheses: (1) no association with either protein or bladder cancer (PH0), (2) 1 causal SNP merely for the protein (PH1), (3) 1 causal SNP merely for bladder cancer (PH2), (4) 2 separate causal SNPs for the protein and bladder cancer (PH3), and (5) a single causal SNP for both protein and bladder cancer (PH4). The prior probabilities for the causal SNP linked to the protein only (p1), bladder cancer only (p2), and both traits (p12) were set as 1 × 10^−4^, 1 × 10^−4^, and 1 × 10^−5^. PH4 greater than 0.8 was considered strong evidence of colocalization. Summary statistics for the proteins were obtained from the deCODE study.^26^ Analyses were conducted using R package coloc.

## Results

### GWAS, TWAS, and enrichment analyses

After quality control, GWAS data for 6984 bladder cancer case patients and 708 432 control individuals of European ancestry were analyzed. The number of case patients and control individuals in each study along with descriptive data on age and sex are provided in Table S3. The genomic inflation statistic was lower than 1.05 in each study (1.045 in UK Biobank, 1.046 in FinnGen, and 1.012 in SIMPLER), indicating negligible inflation due to population stratification. We identified 17 genomic loci, each containing multiple SNPs that attained genome-wide significance (Table S4). These loci included 14 previously reported bladder cancer-associated genes (eg, *GSTM1* and *NAT2*) and 3 novel loci near *MSX2*, *RAPGEF5*, and *MIPOL1* genes (Table 1). Overall, there was little evidence of heterogeneity (*P*_het_>0.05) between the lead SNP-bladder cancer association estimates across studies, except for 2 previously reported loci with moderate heterogeneity (Table 1).

**Table 1.** Genomic loci associated with bladder cancer in the present GWAS meta-analysis.

SNP	Chr	Position	Nearest gene(s)	EA	OA	EAF	OR	95% CI	*P*	***n*** ^a^	***P*_het_** ^b^
**Previously reported loci**											
rs140584594	1	110232983	*GSTM1, GSTM2*	A	G	0.27	0.78	0.74 to 0.83	8.45E-20	1	NA
rs17863783	2	234602277	*UGT1A cluster*	T	G	0.03	0.63	0.56 to 0.71	2.37E-13	3	0.097
rs7628595	3	189618496	*TP63*	T	G	0.25	0.88	0.85 to 0.92	1.34E-09	3	0.425
rs13131466	4	1761543	*TACC3, FGFR3*	T	C	0.78	0.88	0.84 to 0.91	3.30E-10	3	0.527
rs10069690	5	1279790	*TERT, CLPTM1L*	T	C	0.27	0.84	0.80 to 0.87	2.54E-18	3	0.043
rs4646249	8	18260431	*NAT2*	T	G	0.30	0.90	0.86 to 0.93	1.94E-08	3	0.595
rs4075596	8	81988389	*PAG1*	T	C	0.47	0.88	0.85 to 0.91	3.28E-13	3	0.356
rs10094872	8	128719884	*CASC11, MYC*	A	T	0.60	0.80	0.77 to 0.83	2.77E-36	3	0.524
rs2976394	8	143763622	*PSCA*	T	C	0.46	1.18	1.14 to 1.22	8.86E-22	3	0.349
rs11041540	11	1885026	*LSP1, TNNT3*	A	T	0.87	0.85	0.81 to 0.90	5.03E-10	3	0.479
rs9549330	13	113648404	*MCF2L*	T	G	0.28	1.12	1.08 to 1.17	2.05E-08	2	0.578
rs3819177	18	43316110	*SLC14A1*	T	C	0.52	1.11	1.08 to 1.15	7.95E-10	3	0.790
rs8102137	19	30296853	*CCNE1*	T	C	0.66	0.89	0.86 to 0.92	3.22E-10	3	0.019
rs5750711	22	39341957	*APOBEC3A*	T	C	0.61	1.15	1.11 to 1.19	5.75E-14	3	0.239
**Novel loci**											
rs17779033	5	173849901	*RP11-267A15.1, MSX2*	T	C	0.59	1.11	1.07 to 1.15	3.65E-09	3	0.627
rs35675999	7	22397515	*RAPGEF5, STEAP1B*	A	G	0.21	0.88	0.84 to 0.92	3.17E-09	3	0.904
rs1814004	14	37976889	*MIPOL1*	T	C	0.28	1.12	1.08 to 1.16	4.11E-09	3	0.821

Abbreviations: Chr = chromosome; CI = confidence interval; EA = effect allele; EAF = effect allele frequency; NA = not available; OA = other allele; OR = odds ratio; SNP = single-nucleotide polymorphism.

a Number of studies included in the meta-analysis; 2 SNPs were not available in all 3 studies.

b *P*-value for heterogeneity across studies based on Cochran’s *Q* test.

**Figure 1.**
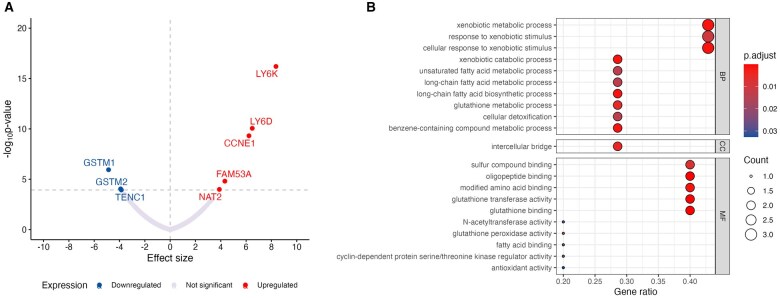
Results of transcriptome-wide association study and Kyoto Encyclopedia of Genes and Genomes (KEGG) enrichment analysis. (**A**) Genes associated with bladder cancer following multiple testing correction (false discovery rate [FDR]) in the transcriptome-wide association study using data from The Cancer Genome Atlas Program, which includes 380 patients with bladder urothelial carcinoma. (**B**) Results of KEGG enrichment analysis, with pathways selected based on an FDR threshold of <0.05.

In the TWAS analysis, we identified 8 genes statistically significantly associated with bladder cancer after FDR correction (Figure 1). Of these, *TENC1* at chromosome 12 (best expression quantitative trait loci rs7315980) was not mapping to the GWAS identified bladder cancer loci. The 8 genes were enriched for pathways related to metabolic and catabolic processes of xenobiotics, long-chain fatty acid metabolic and biosynthetic processes, cellular detoxification, glutathione transferase, N-acetyltransferase, and antioxidant activities (Figure 1).

### MR analysis of proteins

**Figure 2.**
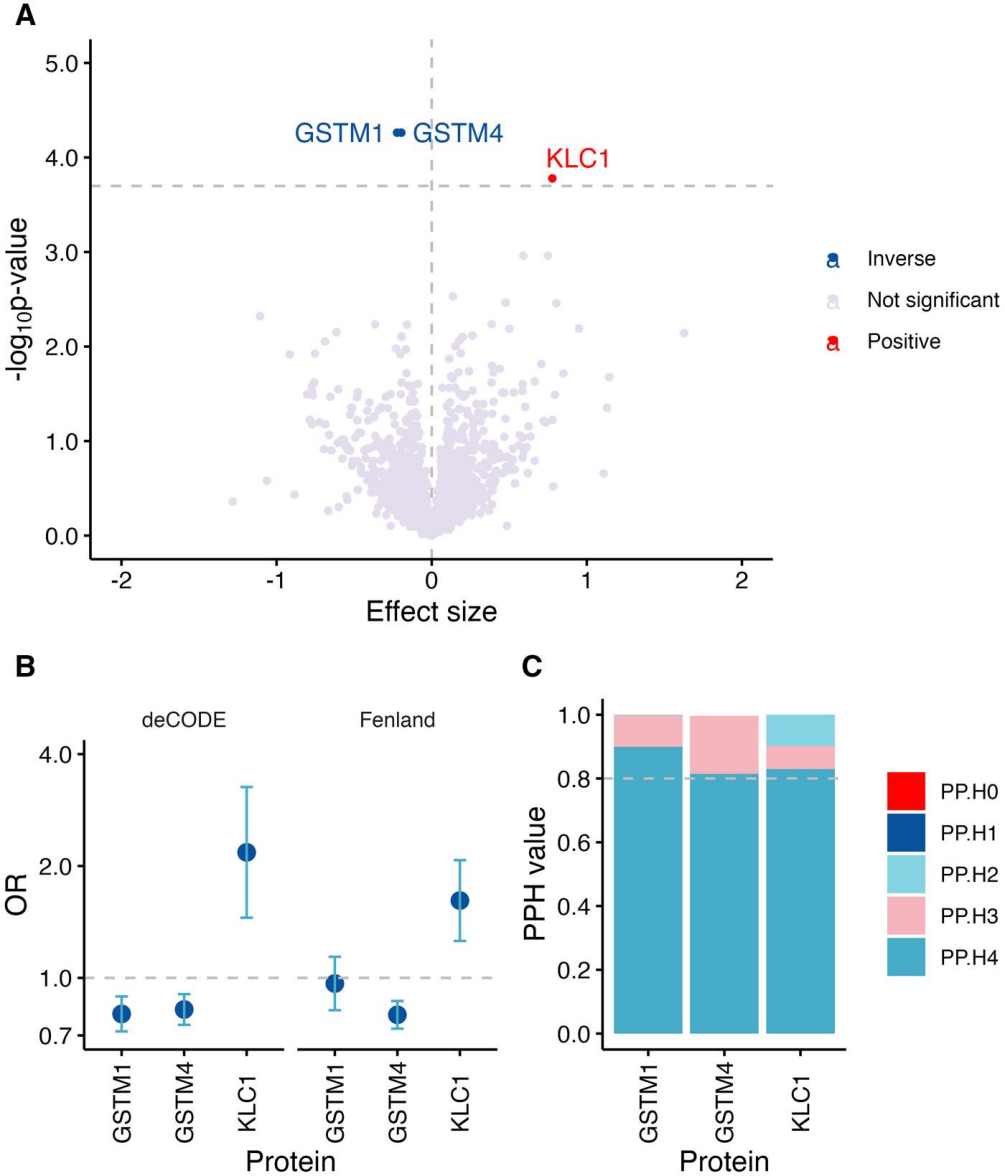
Mendelian randomization (MR) and colocalization results for plasma proteins associated with bladder cancer risk. (**A**) Results of the protein-wide MR analysis of bladder cancer. The associations labeled are significant after correction for false discovery rate (FDR). (**B**) MR associations of GSTM1, GSTM4, and KLC1 with bladder cancer, based on protein data from 2 independent studies (deCODE for discovery and Fenland for replication). (**C**) Results of colocalization analysis using protein data from deCODE, with associations showing strong support for colocalization, as indicated by the Posterior Probability of Colocalization (PP.H4) > 0.8. Abbreviations: GSTM1=glutathione *S*-transferase Mu 1; GSTM4=glutathione *S*-transferase Mu 4; KLC1=kinesin light chain 1; OR=odds ratio.

The discovery analysis of 1793 plasma proteins with a *cis*-pQTL from the deCODE study, which was also available in our bladder cancer GWAS, showed that higher plasma levels of glutathione *S*-transferase mu 1 (GSTM1) and mu 4 (GSTM4), instrumented by the same variant close to *GSTM1*, were associated with a reduced risk of bladder cancer (Figure 2 and Table S5). Higher plasma kinesin light chain 1 (KLC1) was associated with a statistically nonsignificant increased risk of bladder cancer after FDR correction (Figure 2). The associations of GSTM4 and KLC1 but not GSTM1 with bladder cancer risk were replicated (FDR < 0.1) using the corresponding *cis*-pQTLs from the Fenland study (Figure 2 and Table S5). For all 3 proteins (GSTM1, GSTM4, and KLC1), there was strong evidence of colocalization (ie, PH4 ≥ 0.8), indicating that the protein and bladder cancer share a joint causal variant (Figure 2). All colocalization results are shown in Table S6.

### MR analyses of modifiable factors

**Figure 3.**
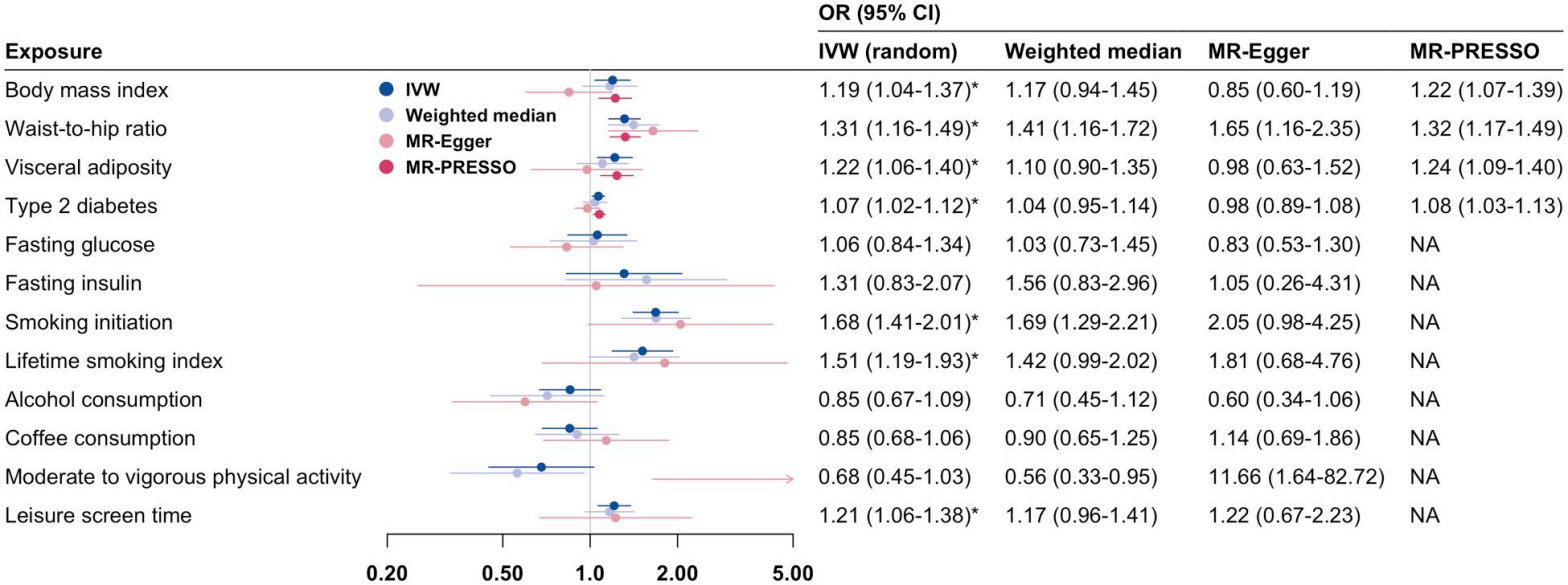
Mendelian randomization analysis of the associations of genetically predicted modifiable factors with bladder cancer risk. Odds ratios (OR) are scaled per SD increase in genetically predicted body mass index, waist-to-hip ratio, visceral adiposity, smoking initiation (prevalence), lifetime smoking index (1 SD increase is equivalent to an individual smoking 20 cigarettes a day for 15 years and stopping 17 years ago, or an individual smoking 60 cigarettes a day for 13 years and stopping 22 years ago), alcohol consumption (log-transformed alcoholic drinks/week), and leisure screen time, per 50% increase in coffee consumption, per log-odds increase in prevalence of genetic liability to type 2 diabetes and moderate-to-vigorous physical activity (active vs inactive), per 1 mmol/L increase in genetically predicted fasting glucose, and per 1 log-transformed picomoles per liter increase in genetically predicted fasting insulin. ^*^Statistically significant association after multiple testing corrections using the false discovery rate (FDR). Abbreviations: CI=confidence interval; IVW=inverse variance weighted (main method); NA=not available; OR=odds ratio.

In univariable MR analysis, genetically predicted higher body mass index, waist-to-hip ratio, visceral adiposity, lifetime smoking index, leisure screen time, and genetic liability to smoking initiation and type 2 diabetes were associated with higher odds of bladder cancer after adjustment for multiple testing (Figure 3). The associations for waist-to-hip ratio, smoking, and leisure screen time were consistent in sensitivity analyses, whereas the associations for body mass index, visceral adiposity, and type 2 diabetes were less stable (Figure 3). There was no statistically significant association between the other studied modifiable factors and bladder cancer risk in the main analysis, and the findings were inconsistent across the sensitivity analyses (Figure 3).

Multivariable MR analysis of various combinations of potential risk factors revealed independent associations between genetically predicted waist-to-hip ratio and smoking and bladder cancer risk (Table S7). The associations between other modifiable factors and bladder cancer observed in the univariable MR analysis did not remain in the multivariable MR analysis. For example, the associations for genetically predicted body mass index and type 2 diabetes did not persist after adjustment for genetically predicted waist-to-hip ratio and the association for genetically predicted leisure screen time did not persist after adjustment for genetic liability to smoking initiation (Table S7).

## Discussion

This GWAS meta-analysis, comprising data from over 700 000 individuals uncovered 17 bladder cancer susceptibility loci. Our study confirmed a key role of detoxification processes, glutathione *S*-transferases, smoking, and abdominal obesity in bladder cancer etiology.

The 24 bladder cancer susceptibility loci identified in a recent GWAS meta-analysis including 13 790 case patients and 343 502 control individuals^22^ were replicated in our nonoverlapping GWAS sample, although not all lead SNPs in previous GWAS attained genome-wide significance in our GWAS (Table S8). In our meta-analysis, the associations of the identified 3 novel loci (5:173849901, 7:22397515, and 14:37976889) with bladder cancer risk were similar across studies. The novel locus on chromosome 5 is located near *MSX2*, encoding muscle segment homeobox 2, which is a biomarker for the diagnosis and prediction of tumorigenesis in some tumors.^31^ The genomic region on chromosome 7 is located near *RAPGEF5*, which regulates Rap proteins, and mutations in this gene have been linked to several diseases, including certain cancers such as renal cell carcinoma.^32^ Finally, the novel locus on chromosome 14 maps to the *MIPOL1* tumor suppressor gene, previously shown to be linked to certain cancers.^33^ Our TWAS identified an additional gene, namely *TENC1* (also known as *C1TEN*, *C1-TEN*, and *TNS2*), not mapping to the genome-wide statistically significant bladder cancer loci identified in the present or previous GWAS.^22^ Further studies are required to understand the role of these previously unreported genes in bladder carcinogenesis.

Our analyses showed a consistent association between GSTM1 and bladder cancer risk. The glutathione *S*-transferase family comprises important phase II antioxidant enzymes that are involved in the detoxification of many endogenous and exogenous substances.^34^ For example, carcinogenic metabolites of environmental pollutants and tobacco smoke (eg, polycyclic aromatic hydrocarbon diol-epoxides) are detoxified by members of the glutathione *S*-transferase family.^34^ Available evidence indicates that homozygous for the GSTM1-null genotype carry a higher risk for bladder cancer, particularly if exposed to certain toxicants, like asbestos, rubber, and chlorinated solvents.^34^ In addition to GSTM1, our GWAS meta-analysis corroborates a strong association with the *NAT2* locus. *NAT2* encodes the enzyme N-acetyltransferase 2, which like the glutathione *S*-transferases is involved in phase II detoxification of carcinogens such as aromatic amines from tobacco smoke and many other compounds. Previous studies have observed an interaction between *NAT2* and smoking^22^^,^^35^ in relation to bladder cancer risk. It has also been estimated that a slow acetylation phenotype in both *GSTM1* and *NAT2* may account for up to 31% of bladder cancer case patients.^36^

Glutathione (a tripeptide and the most abundant antioxidant in human cells^37^) is a cofactor for glutathione *S*-transferases in the detoxification of xenobiotics and drugs. The potential role of glutathione supplementation in bladder cancer prevention is unknown. High levels of glutathione might be detrimental during cancer therapy, as glutathione could protect cancer cells from apoptosis induced by oxidative stress.^37^ Moreover, glutathione depletion in cancer cells may increase their susceptibility to cancer therapy.^37^

Our proteome-wide MR suggested a possible association between higher KLC1 levels and increased risk of bladder cancer. The role of this protein in cancer remains unclear, and further research on the potential involvement of this protein in bladder cancer is required.

Our MR study, together with previous observational and experimental data^38^ triangulated the evidence for a causal effect of smoking on bladder carcinogenesis. Alcohol and coffee consumption are other lifestyle factors associated with certain cancers. A meta-analysis of 16 case-control and 3 cohort studies found no association between self-reported alcohol consumption and bladder cancer risk.^12^ A European cohort study of nearly half a million individuals observed associations between high alcohol consumption and increased bladder cancer risk in men and smokers.^13^ Similarly, another pooled analysis involving over half a million participants found that high coffee consumption (>4 cups/day) was associated with an increased risk of bladder cancer only in male smokers.^14^ Our MR analyses provided no support for the positive associations between genetically predicted higher alcohol or coffee consumption and bladder cancer risk. If anything, the association was inverse. Thus, the previously observed associations between alcohol and coffee consumption and bladder cancer risk in male smokers may be caused by residual confounding from smoking.

We found evidence of a causal association between greater adiposity, particularly abdominal obesity, measured as waist-to-hip ratio, and an increased risk of bladder cancer. Observational studies of the association between body mass index, reflecting overall adiposity, and risk of bladder cancer showed either no association,^39^ a positive association in men but an inverse association in women,^40^ or an association for high-grade bladder cancer only.^41^ One study observed an increased risk of bladder cancer only at the higher end of body mass index and waist-to-hip ratio.^42^ A possible explanation for these inconsistent findings is residual confounding from smoking.

Our MR results provided no evidence of a causal association between fasting glucose or fasting insulin levels and the risk of bladder cancer. While we observed an association between genetic liability to type 2 diabetes and bladder cancer risk in the univariable MR analysis, the association did not remain after adjustment for genetically predicted waist-to-hip ratio. The positive association between fasting blood glucose^1^ and type 2 diabetes^7^^,^^8^ found in previous observational studies may be related to residual confounding from obesity or an adverse effect of diabetes medications on bladder cancer risk.

A few observational studies have found that moderate to vigorous physical activity is associated with a reduced risk of bladder cancer,^9^^,^^10^ whereas excess leisure sitting time is associated with an increased risk.^9^ Our MR analysis corroborates the direction of these associations, but the analysis of moderate to vigorous physical activity lacked power and the main MR estimate did not retain statistical significance after multiple testing corrections. Moreover, the association between genetically predicted leisure screen time and bladder cancer risk in our study did not persist in the multivariable MR analysis adjusted for smoking initiation, indicating that this association was driven by genetic confounding (pleiotropy).

The strengths of this study include the large sample size and appraisal of the causal role of hundreds of plasma proteins and many potentially modifiable risk factors for bladder cancer. Our study has several limitations. First, the study sample was restricted to individuals of European ancestries (to reduce bias due to population stratification), which reduced the assignability of our findings to non-European populations. Another shortcoming is that we were unable (due to limited statistical power) to investigate gene–sex and gene–smoking interactions because of the lack of individual phenotype data in FinnGen, which contributed to a third of all case patients in our GWAS meta-analysis.

In conclusion, this study supports a key role for detoxification processes, particularly glutathione *S*-transferases, in bladder cancer. Smoking and abdominal obesity were identified as major modifiable risk factors for bladder cancer.

## Supplementary Material

pkaf014_Supplementary_Data

## Data Availability

Summary level data for this GWAS meta-analysis and the SIMPLER cohorts are available at the OSF data repository via the link https://osf.io/yzwnp/.
