# Supplementary material for: Genome-wide association study and Mendelian randomization analyses reveal insights into bladder cancer etiology
Source: JNCI Cancer Spectr. 2025 Feb 3;9(2):pkaf014. doi: 10.1093/jncics/pkaf014 (PMC11950924; doi:10.1093/jncics/pkaf014)
Supplement: pkaf014_Supplementary_Data [file pkaf014_supplementary_data.zip › Supplementary_Material.pdf]

# Supplementary Material

## Supplementary Methods

### *UK Biobank*

UK Biobank is a research initiative aimed to support scientific breakthroughs to enhance human health. The study recruited approximately 500 000 UK adults, aged between 40 and 69 years, from 2006 to 2010 and collected genetic and other phenotype data. To form the sample population for our GWAS analysis, we removed individuals of non-British ancestry, uncertain sex, without genetic data or high missingness on genotype, marked deviations in heterozygosity, and related individuals (third-degree or closer). We excluded single-nucleotide polymorphisms with a missingness rate >10%, a Hardy-Weinberg equilibrium *P*-value $<1\times10^{-6}$, or a minor allele frequency <0.01. Cases of bladder cancer were ascertained from inpatient and outpatient records using the International Classification of Diseases (ICD) codes 188 for ICD-8 and ICD-9 and C67 for ICD-10. After quality control, the analysis included 3961 bladder cancer cases (incident and prevalent with a primary or secondary diagnosis) and 368 773 controls without a bladder cancer diagnosis. To obtain the genetic association estimates, we used logistic mixed model applied in the REGENIE v2.0 software[16] and adjusted for age, sex, genotyping batch, and the first ten genetic principal components. All participants in the UK Biobank cohort had provided written informed consent. The cohort was approved by the Northwest Multi-centre Research Ethics Committee.

### *FinnGen*

FinnGen is an ongoing project that combines genotype data and national health databases with the aim to provide new knowledge on the genetics of human diseases.[17] Cases of bladder cancer were ascertained through inpatient, outpatient, and social insurance reimbursement records and defined by the ICD-8 and ICD-9 code 188 and ICD-10 code C67, as primary or secondary diagnosis and included both incident and prevalent diagnoses. We used the 10th release of the FinnGen summary statistics data, which included 2193 bladder cancer cases and 314 193 non-cases. Excluded from the analysis were individuals of uncertain sex, related individuals, and those with non-Finnish genetic origin, high genotype missingness, or excess heterozygosity. SNPs with a missingness rate >2%, a Hardy-Weinberg equilibrium *P*-value $<1\times10^{-6}$, or a minor allele count <3 were omitted. The genetic association estimates were adjusted for age, sex, genotyping batch, and the first 10 genetic principal components. Participants in FinnGen provided written informed consent, and the study was approved by the Finnish Institute for Health and Welfare, the Digital and Population Data Service Agency, the Social Insurance Institution, and Statistics Finland.[17]

### *SIMPLER*

The Swedish Infrastructure for Medical Population-based Life-course and Environmental Research (SIMPLER) brings together two population-based cohort studies of Swedish adults born between 1914 and 1952 and residing in Uppsala, Västmanland, and Örebro counties in central Sweden (www.simpler4health.se/w/sh/en). In 1997, participants provided detailed information on their diet, lifestyle behaviors, and other risk factors for chronic diseases. DNA was obtained either from saliva samples collected in the main cohorts or from blood samples collected in two clinical subcohorts. Individuals of non-European ancestry, high genotype missingness, or excess heterozygosity were excluded from the GWAS analysis. Omitted were also SNPs with a missingness rate >2%, a Hardy-Weinberg equilibrium $P$-value $<1\times10^{-7}$ or allele frequency difference >0.15 compared with 1000G/HRC. Bladder cancer cases were ascertained through the Swedish National Cancer Register and defined by the ICD-8 and ICD-9 code 188 and ICD-10 code C67. After quality control, the analysis included a total of 830 bladder cancer cases as primary diagnosis and diagnosed before the data collection in 1997 (prevalent cases) or during follow-up until 2021 (incident cases), and 25 466 individuals without bladder cancer. The GWAS analysis was conducted in PLINK 2 and adjustments were made for sex, age (at 1997-01-01), and the first six genetic principal components. Cohort participants have provided informed consent, and data collection and genetic analyses in the cohorts have been approved by the Swedish Ethical Review Authority. The statistical analysis and data handling of SIMPLER were enabled by resources in project simp2023021 provided by the National Academic Infrastructure for Supercomputing in Sweden (NAISS) at the Uppsala Multidisciplinary Center for Advanced Computational Science (UPPMAX), partially funded by the Swedish Research Council through grant agreement no. 2022-06725.

**Supplementary Table 1.** Information on the GWAS meta-analyses used to obtain genetic instruments for modifiable factors

| **Modifiable factor** | **No. SNPs*** | ***F* statistic** | **Unit** | **PubMed ID** | **Sample size or cases/controls** | **Ancestry** |
|---|---|---|---|---|---|---|
| Body mass index | 311 | 89 | SD | 30239722 | 806 834 | European |
| Waist-to-hip ratio | 578 | 56 | SD | 30239722 | 697 734 | European |
| Visceral adiposity | 294 | 50 | SD | 31501611 | 397 170 | European |
| Type 2 diabetes | 497 | 84 | Log-odds in prevalence of type 2 diabetes | 32541925 | 228 499 cases/ 1 178 783 controls | European |
| Fasting glucose | 69 | 124 | 1 mmol/L | 34059833 | Up to 196 991 | European |
| Fasting insulin | 38 | 52 | 1 log-transformed picomoles per L | 34059833 | Up to 196 991 | European |
| Smoking initiation | 346 | 36 | SD in prevalence of smoking initiation | 36477530 | 2 669 029 | European |
| Lifetime smoking | 126 | 48 | SD | 31689377 | 462 690 | European |
| Alcohol consumption | 113 | 57 | SD increase of log-transformed alcoholic drinks | 36477530 | 2 428 851 | European |
| Coffee consumption | 12 | 176 | 50% change | 31046077 | 375 833 | European |
| Moderate to vigorous physical activity | 16 | 38 | Being active vs. inactive | 36071172 | 298 506 | European |
| Leisure screen time | 132 | 39 | SD | 36071172 | 526 725 | European |

*Number of SNPs used as instrumental variables for the modifiable factor. Abbreviations: SD, standard deviation; SNPs, single-nucleotide polymorphisms.

**Supplementary Table 2.** Genetic variants used as instrumental variables for the modifiable factors in Mendelian randomization analysis.

- *See separate Excel file: Supplementary Table 2.*

**Supplementary Table 3.** Number of cases and controls and characteristics of studies included in bladder cancer GWAS meta-analysis

| Study | No. Cases | No. Controls | Age, median | Women, % |
|---|---|---|---|---|
| FinnGen R10 | 2193 | 314 193 | 60 | 56,5 |
| SIMPLER | 686 | 17 197 | 58 | 22 |
| SIMPLER-Västerås subcohort | 129 | 4 699 | 55 | 37 |
| SIMPLER-Uppsala subcohort | 15 | 3 570 | 56 | 100 |
| UK Biobank | 3961 | 368 773 | 58 | 54 |

**Supplementary Table 4.** Genomic region associated with bladder cancer in the present genome-wide association study meta-analysis.

- *See separate Excel file: Supplementary Table 4*.

**Supplementary Table 5.** Associations of plasma proteins with bladder cancer risk using genetic instruments from the deCODE study (discovery analysis) and the corresponding associations using genetic instruments from the Fenland study (replication analysis).

- *See separate Excel file: Supplementary Table 5*.

**Supplementary Table 6.** Colocalization results based on summary statistics data for the proteins from the deCODE study

| PP.H0.abf | PP.H1.abf | PP.H2.abf | PP.H3.abf | PP.H4.abf | SeqId | Protein |
|---|---|---|---|---|---|---|
| 2.01034171915957×10^-197 | 1.1254630397691×10^-195 | 0,001787732 | 0,09918477 | 0,899027498 | 15395_15 | GSTM1 |
| 1.85803790573667×10^-19 | 1.38199240581452×10^-19 | 0,097993802 | 0,072056987 | 0,829949211 | 12656_1 | KLC1 |
| 1.99609559983053×10^-197 | 1.11748753957818×10^-195 | 0,003268649 | 0,182176422 | 0,814554928 | 18895_54 | GSTM4 |
| 4.96937432367657×10^-196 | 1.47571149923841×10^-195 | 0,062035897 | 0,183468066 | 0,754496037 | 3066_12 | |
| 2.15408710573627×10^-19 | 2.72932546967895×10^-20 | 0,340010629 | 0,042463353 | 0,617526018 | 5307_12 | |
| 1.82364938067491×10^-27 | 3.60710773114685×10^-28 | 0,365820638 | 0,071795519 | 0,562383842 | 5131_15 | |
| 2.06712033420123×10^-15 | 1.59528495975857×10^-15 | 0,300321769 | 0,231302747 | 0,468375484 | 8957_72 | |
| 1.02552759564891×10^-12 | 3.50895610123276×10^-13 | 0,435349219 | 0,148543443 | 0,416107337 | 2737_22 | |
| 4.40916403418535×10^-30 | 7.48839629978429×10^-31 | 0,51732446 | 0,087465676 | 0,395209864 | 15368_3 | |
| 1.59583557417183×10^-38 | 3.33636908025503×10^-39 | 0,511934896 | 0,106647388 | 0,381417716 | 13107_9 | |
| 0,0075805 | 0,000956651 | 0,54946374 | 0,068968739 | 0,37303037 | 13697_51 | |
| 1.95289482806245×10^-192 | 5.24836160936481×10^-193 | 0,513672996 | 0,137699853 | 0,348627151 | 5634_39 | |
| 2.98838094804842×10^-196 | 3.21694889387893×10^-196 | 0,329608009 | 0,354502368 | 0,315889623 | 17692_2 | |
| 0,000173506 | 5.01227637041007×10^-05 | 0,545261225 | 0,15721927 | 0,297295877 | 10075_75 | |
| 3.12678163371722×10^-18 | 4.7705709637735×10^-19 | 0,626901813 | 0,095369487 | 0,277728699 | 13642_90 | |
| 1.15755784315424×10^-44 | 3.14929406255915×10^-45 | 0,570790787 | 0,15501723 | 0,274191983 | 12432_23 | |
| 8.68269058437542×10^-196 | 1.81211748062563×10^-196 | 0,620030479 | 0,129152397 | 0,250817124 | 17138_8 | |
| 7.49188339860462×10^-131 | 9.55931426718315×10^-132 | 0,665517847 | 0,08466734 | 0,249814812 | 3519_3 | |
| 2.19727331989837×10^-94 | 2.50171359343111×10^-95 | 0,701357738 | 0,079634334 | 0,219007928 | 7841_84 | |
| 7.26957419772308×10^-181 | 2.15631319057288×10^-181 | 0,603460546 | 0,178781713 | 0,21775774 | 15533_97 | |
| 7.52595291815636×10^-25 | 1.32191011171868×10^-25 | 0,665991544 | 0,116762087 | 0,217246369 | 3806_55 | |
| 5.96842736627691×10^-11 | 7.67714598994719×10^-12 | 0,693979652 | 0,089049142 | 0,216971206 | 10015_119 | |
| 9.33440759742847×10^-07 | 8.19312152083562×10^-08 | 0,734734918 | 0,064289164 | 0,200974902 | 14112_40 | |
| 8.8253584711645×10^-08 | 7.56971731351731×10^-09 | 0,755030593 | 0,064580359 | 0,180388952 | 10668_5 | |
| 3.14636082881499×10^-189 | 3.38335478768264×10^-190 | 0,741033805 | 0,079505624 | 0,179460572 | 11424_4 | |
| 1.39515676830154×10^-194 | 2.91993607632303×10^-195 | 0,68290004 | 0,14275041 | 0,17434955 | 12386_11 | |
| 2.74909563604669×10^-09 | 3.2037469519491×10^-10 | 0,740851528 | 0,08616453 | 0,172983939 | 4533_76 | |
| 9.38349487087828×10^-09 | 1.60309957136906×10^-09 | 0,714567688 | 0,121915001 | 0,1635173 | 11382_5 | |
| 1.53845397223192×10^-20 | 2.64004874697929×10^-21 | 0,714810517 | 0,122501665 | 0,162687818 | 13438_115 | |
| 1.51104209029528×10^-05 | 4.18783756743735×10^-06 | 0,657481339 | 0,182059835 | 0,160439527 | 5598_3 | |
| 1.20692370614487×10^-19 | 2.76799290424618×10^-20 | 0,684698162 | 0,15687218 | 0,158429658 | 12395_86 | |
| 7.92210786297198×10^-195 | 4.01346128321857×10^-195 | 0,562395279 | 0,28476523 | 0,152839491 | 8402_22 | |
| 8.47581918717368×10^-37 | 9.16447209341416×10^-38 | 0,764869775 | 0,082548896 | 0,152581329 | 5604_30 | |
| 0,000411319 | 6.64433992674565×10^-05 | 0,73428321 | 0,118467542 | 0,146771486 | 2968_61 | |
| 4.0614501796054×10^-06 | 4.57794084379199×10^-07 | 0,775588289 | 0,087284787 | 0,137122404 | 5109_24 | |
| 1.02477602177334×10^-161 | 1.12017496454287×10^-162 | 0,779411793 | 0,08506139 | 0,135526816 | 2590_69 | |
| 2.01040370182241×10^-05 | 3.01504930654886×10^-06 | 0,754572743 | 0,113032662 | 0,132371476 | 17712_7 | |
| 2.4919517781489×10^-57 | 4.89554056423616×10^-58 | 0,726518443 | 0,142596616 | 0,13088494 | 17148_7 | |
| 1.71829655290976×10^-19 | 1.49932287561783×10^-20 | 0,803640608 | 0,069996384 | 0,126363008 | 2960_66 | |

| | | | | | |
|---|---|---|---|---|---|
| 7.67706231460186×10^-189 | 1.07885578818301×10^-189 | 0,766881622 | 0,107644223 | 0,125474155 | 2571_12 |
| 1.57234184009438×10^-137 | 1.74195569216604×10^-138 | 0,790551091 | 0,087461064 | 0,121987845 | 17456_53 |
| 2.12074760281315×10^-194 | 2.44089892892684×10^-195 | 0,789194921 | 0,090713208 | 0,12009187 | 6649_51 |
| 2.46906214834997×10^-64 | 2.73540826425619×10^-65 | 0,79308991 | 0,087745156 | 0,119164934 | 8983_7 |
| 6.83593839779316×10^-25 | 1.08660443068651×10^-25 | 0,763048454 | 0,121174349 | 0,115777197 | 7140_1 |
| 1.25699074640785×10^-11 | 4.91630169353466×10^-12 | 0,636255409 | 0,248735156 | 0,115009435 | 13534_20 |
| 5.51353617936385×10^-194 | 3.43186748629209×10^-195 | 0,844204214 | 0,052443632 | 0,103352154 | 5676_54 |
| 1.34999077074887×10^-14 | 1.61534863824329×10^-15 | 0,805157456 | 0,096243541 | 0,098599003 | 13450_49 |
| 0,000114271 | 1.01645714458799×10^-05 | 0,828490199 | 0,073597376 | 0,097787989 | 10534_40 |
| 2.29659036609888×10^-194 | 4.36727618197196×10^-195 | 0,7614424 | 0,144704684 | 0,093852915 | 4430_44 |
| 5.42825084648669×10^-21 | 1.35319950939557×10^-21 | 0,725462409 | 0,18075552 | 0,093782071 | 10722_13 |
| 3.53456719830631×10^-39 | 2.41301436991924×10^-39 | 0,539604692 | 0,368290728 | 0,092104581 | 13578_98 |
| 2.41225753205227×10^-195 | 4.50652591534631×10^-196 | 0,765881067 | 0,142989069 | 0,091129864 | 3220_40 |
| 3.90219018276683×10^-31 | 7.86885906863929×10^-32 | 0,7566496 | 0,152489326 | 0,090861074 | 2617_56 |
| 7.71840619476862×10^-196 | 2.07072089383605×10^-196 | 0,719038649 | 0,19281805 | 0,088143302 | 7926_13 |
| 3.5330346731396×10^-106 | 6.89040160604735×10^-107 | 0,764437659 | 0,149000072 | 0,086562269 | 2652_15 |
| 1.50473393539415×10^-41 | 2.40040957870445×10^-42 | 0,791560341 | 0,126190508 | 0,082249151 | 2851_63 |
| 3.97083904146544×10^-14 | 4.15446145573142×10^-15 | 0,833463386 | 0,087121085 | 0,079415529 | 15585_304 |
| 1.51259104320911×10^-108 | 1.46603765757758×10^-109 | 0,839834794 | 0,081319854 | 0,078845352 | 5852_6 |
| 8.90759718282903×10^-07 | 1.04601604367373×10^-07 | 0,824630551 | 0,096757468 | 0,078610986 | 12347_29 |
| 0,010451061 | 0,001012941 | 0,829632026 | 0,080331251 | 0,078572722 | 5339_49 |
| 2.84559479642265×10^-176 | 6.7028182196383×10^-176 | 0,274767548 | 0,647138765 | 0,078093687 | 9005_16 |
| 9.67357515513808×10^-57 | 2.07626296838155×10^-57 | 0,759912402 | 0,163024785 | 0,077062814 | 8480_29 |
| 2.65411939471097×10^-195 | 1.85008909119241×10^-195 | 0,550153891 | 0,383425639 | 0,06642047 | 3181_50 |
| 1.29173100346664×10^-194 | 4.45179153309224×10^-195 | 0,697275326 | 0,240244859 | 0,062479815 | 6551_94 |
| 1.62408368413508×10^-47 | 2.49264134246319×10^-48 | 0,817133394 | 0,125356007 | 0,057510599 | 5638_23 |
| 6.84677185617867×10^-195 | 2.68570210029593×10^-195 | 0,678393144 | 0,266049686 | 0,055557169 | 8814_33 |
| 3.13266282339508×10^-195 | 4.90435222608143×10^-196 | 0,816753327 | 0,127811693 | 0,05543498 | 5737_61 |
| 9.66086031782817×10^-136 | 1.55465232714678×10^-136 | 0,814355244 | 0,130993644 | 0,054651112 | 10440_26 |
| 2.25991126871399×10^-138 | 2.35880127896297×10^-139 | 0,856285726 | 0,089321145 | 0,054393129 | 19561_216 |
| 1.98768181515895×10^-50 | 2.29283486613804×10^-51 | 0,848970784 | 0,097877503 | 0,053151713 | 4911_49 |
| 0,000140121 | 1.22316304349027×10^-05 | 0,872845491 | 0,076142972 | 0,050859185 | 4314_12 |
| 7.67720389593881×10^-11 | 8.41570256790334×10^-12 | 0,855434885 | 0,09372139 | 0,050843725 | 9765_4 |
| 3.3049286750838×10^-38 | 3.34066805747198×10^-39 | 0,86224646 | 0,087106429 | 0,05064711 | 12697_30 |
| 5.58123054631403×10^-195 | 8.38830485296738×10^-196 | 0,825549193 | 0,124025408 | 0,050425399 | 5736_1 |
| 2.19388369666566×10^-195 | 2.59215439796881×10^-196 | 0,850573921 | 0,100449465 | 0,048976613 | 7871_16 |
| 9.18576906392966×10^-43 | 2.41740236512417×10^-43 | 0,753441295 | 0,198233469 | 0,048325237 | 2602_2 |
| 5.67413835863909×10^-196 | 4.92037426556288×10^-197 | 0,876334862 | 0,075944355 | 0,047720783 | 15584_9 |
| 7.67528800028983×10^-195 | 2.04066658330891×10^-195 | 0,752915135 | 0,200134301 | 0,046950564 | 3449_58 |
| 3.43158529066846×10^-05 | 2.27515135944622×10^-06 | 0,896032843 | 0,059362679 | 0,044567887 | 17781_191 |
| 2.39194954238219×10^-195 | 4.20193094130624×10^-196 | 0,815174287 | 0,143159769 | 0,041665945 | 6947_4 |
| 1.1587902103628×10^-194 | 1.98304362406149×10^-195 | 0,820992104 | 0,14045825 | 0,038549646 | 8397_147 |
| 5.49762810046516×10^-10 | 7.78692989249912×10^-10 | 0,398504036 | 0,564410516 | 0,037085446 | 5092_51 |

| | | | | | |
|---|---|---|---|---|---|
| 4.79866976317707×10^-195 | 8.88309923222877×10^-196 | 0,812622694 | 0,150392371 | 0,036984935 | 8766_29 |
| 1.22233520539408×10^-194 | 2.35916883864411×10^-195 | 0,807306656 | 0,155777356 | 0,036915988 | 2515_14 |
| 1.99800052753665×10^-195 | 3.69638095201179×10^-196 | 0,816705977 | 0,151061642 | 0,032232381 | 6391_52 |
| 7.44019712117801×10^-195 | 1.37729855556906×10^-195 | 0,817075672 | 0,151221971 | 0,031702357 | 7787_25 |
| 2.03315895427705×10^-194 | 2.81046973193053×10^-195 | 0,853716623 | 0,11798238 | 0,028300997 | 8759_29 |
| 1.59022892575151×10^-192 | 2.01052146093729×10^-193 | 0,871579964 | 0,110175338 | 0,018244698 | 5660_51 |
| 5.2181590038356×10^-93 | 6.70751829098849×10^-94 | 0,870437293 | 0,11186993 | 0,017692777 | 16916_19 |
| 2.189704773724×10^-196 | 2.56741710894345×10^-197 | 0,879429646 | 0,103095179 | 0,017475175 | 16620_26 |
| 2.90986545961539×10^-195 | 5.31526088031014×10^-196 | 0,831064088 | 0,151787892 | 0,01714802 | 3296_92 |
| 9.46269840191737×10^-49 | 9.46490670893188×10^-50 | 0,894575335 | 0,089462448 | 0,015962217 | 3805_16 |
| 7.00457839602984×10^-195 | 1.75227716017817×10^-195 | 0,792329055 | 0,198200904 | 0,00947004 | 3194_36 |
| 2.02610212650281×10^-194 | 2.48949198815138×10^-193 | 0,074656732 | 0,917306711 | 0,008036557 | 11219_95 |
| 3.86700214385317×10^-144 | 1.06031729509575×10^-141 | 0,003632362 | 0,995979402 | 0,000388236 | 13934_3 |

**Supplementary Table 7.** Multivariable Mendelian randomization analyses of different combinations of potentially modifiable bladder cancer risk factors

| **Waist-to-hip ratio and body mass index** | | | | | |
|---|---|---|---|---|---|
| nSNP = 742 | | | | | |
| **Exposure** | **Beta** | **SE** | **LB** | **UB** | **P-value** |
| Waist-to-hip ratio | 0,253 | 0,073 | 0,11 | 0,397 | 0,001 |
| Body mass index | 0,013 | 0,075 | -0,134 | 0,159 | 0,866 |

| **Waist-to-hip ratio and type 2 diabetes** | | | | | |
|---|---|---|---|---|---|
| nSNPs=886 | | | | | |
| **Exposure** | **Beta** | **SE** | **LB** | **UB** | **P-value** |
| Waist-to-hip ratio | 0,296 | 0,07 | 0,158 | 0,434 | 2,68E-05 |
| Type 2 diabetes | 0,021 | 0,024 | -0,027 | 0,068 | 0,391 |

| **Waist-to-hip ratio and smoking inititation** | | | | | |
|---|---|---|---|---|---|
| nSNPs=796 | | | | | |
| **Exposure** | **Beta** | **SE** | **LB** | **UB** | **P-value** |
| Waist-to-hip ratio | 0,229 | 0,063 | 0,106 | 0,352 | 2,69E-04 |
| Smoking | 0,406 | 0,105 | 0,2 | 0,613 | 1,16E-04 |

| **Waist-to-hip ratio and leisure screen time** | | | | | |
|---|---|---|---|---|---|
| nSNP = 652 | | | | | |
| **Exposure** | **Beta** | **SE** | **LB** | **UB** | **P-value** |
| Waist-to-hip ratio | 0,199 | 0,067 | 0,068 | 0,331 | 0,003 |
| Leisure screen time | 0,216 | 0,079 | 0,06 | 0,371 | 0,007 |

| **Smoking initiation and type 2 diabetes** | | | | | |
|---|---|---|---|---|---|
| nSNPs=722 | | | | | |
| **Exposure** | **Beta** | **SE** | **LB** | **UB** | **P-value** |
| Smoking initiation | 0,458 | 0,101 | 0.260, | 0,656 | 5,75E-06 |
| Type 2 diabetes | 0,049 | 0,022 | 0.006, | 0,092 | 0,024 |

| **Smoking initiation and leisure screen time** | | | | | |
|---|---|---|---|---|---|
| nSNPs=429 | | | | | |
| **Exposure** | **Beta** | **SE** | **LB** | **UB** | **P-value** |
| Smoking initiation | 0,458 | 0,101 | 0,261 | 0,655 | 5,17E-06 |
| Leisure screen time | 0,087 | 0,071 | -0,053 | 0,227 | 0,222 |

**Supplementary Table 8.** Previously reported loci (lead single-nucleotide polymorphism) and their associations with bladder cancer risk in the present GWAS meta-analysis

| | | | | Previous GWAS (Koutros et al. 2023) | | | | Present GWAS (Larsson et al.) | | |
|---|---|---|---|---|---|---|---|---|---|---|
| **SNP** | **Chr** | **Position** | **Nearby gene(s)** | **EA** | **NEA** | **OR*** | **P-value** | **Beta*** | **OR*** | **P-value** |
| GSTM1 composite | 1 | 110229772 | *GSTM1* | (-), G | (+), A | 1,20 | 8,84E-23 | 0,24† | 1,28** | 8.45E-20 |
| rs17863783 | 2 | 234602277 | *UGT1A cluster* | G | T | 1,75 | 9,46E-20 | 0,46 | 1,59 | 2,37E-13 |
| rs10936599 | 3 | 169492101 | *MYNN, TERC* | C | T | 1,10 | 1,16E-06 | 0,06 | 1,06 | 2,37E-03 |
| rs710521 | 3 | 189645933 | *TP63* | T | C | 1,15 | 4,56E-14 | 0,13 | 1,13 | 1,75E-09 |
| rs2896518 | 4 | 1757559 | *TACC3, FGFR3* | A | G | 1,17 | 5,28E-15 | 0,13 | 1,14 | 3,79E-09 |
| rs2242652 | 5 | 1280028 | *CLPTM1L, TERT* | G | A | 1,18 | 4,06E-15 | 0,19 | 1,21 | 8,05E-18 |
| rs6910215 | 6 | 20783394 | *CDKAL1* | C | T | 1,10 | 1,05E-08 | 0,05 | 1,05 | 7,76E-03 |
| rs72826305 | 6 | 21826729 | *CASC15* | C | T | 1,12 | 1,81E-10 | 0,09 | 1,09 | 3,80E-06 |
| rs2125484 | 7 | 155759638 | *LOC389602* | G | A | 1,11 | 1,42E-09 | 0,07 | 1,07 | 0,022 |
| rs1495741 | 8 | 18272881 | *NAT2* | A | G | 1,16 | 6,53E-13 | 0,11 | 1,12 | 1,07E-07 |
| rs5003154 | 8 | 81986953 | *PAG1* | C | T | 1,11 | 1,15E-10 | 0,11 | 1,12 | 2,84E-10 |
| rs10094872 | 8 | 128719884 | *CASC11, MYC* | T | A | 1,24 | 1,20E-37 | 0,22 | 1,25 | 2,77E-36 |
| rs2294008 | 8 | 143761931 | *PSCA* | T | C | 1,14 | 1,31E-15 | 0,17 | 1,18 | 3,00E-21 |
| rs1414253 | 9 | 21755630 | *MTAP/CDKN2A* | A | G | 1,08 | 4,15E-06 | 0,07 | 1,08 | 2,34E-05 |
| rs4743687 | 9 | 106856910 | *SMC2* | C | T | 1,10 | 2,05E-08 | 0,04 | 1,05 | 0,012 |
| rs7076867 | 10 | 71582996 | *COL13A1* | C | T | 1,31 | 5,60E-13 | 0,11 | 1,12 | 0,005 |
| rs907611 | 11 | 1874072 | *TNNT3, LSP1* | A | G | 1,11 | 2,53E-08 | 0,08 | 1,08 | 3,02E-05 |
| rs7937265 | 11 | 1947800 | *TNNT3, LSP1* | G | C | 1,13 | 1,10E-08 | 0,08 | 1,08 | 5,23E-04 |
| rs4907479 | 13 | 113659108 | *MCF2L* | A | G | 1,10 | 7,00E-08 | 0,11 | 1,11 | 8,36E-08 |
| rs10853535 | 18 | 43317547 | *SLC14A1* | C | T | 1,14 | 8,02E-16 | 0,10 | 1,11 | 4,85E-09 |
| rs8102137 | 19 | 30296853 | *CCNE1* | C | T | 1,12 | 1,61E-11 | 0,12 | 1,12 | 3,22E-10 |
| rs411482 | 19 | 49103447 | *SULT2B1-FAM83E* | C | T | 1,13 | 1,16E-12 | 0,05 | 1,05 | 0,015 |
| rs62185668 | 20 | 10961935 | *gene desert* | A | C | 1,11 | 9,39E-09 | 0,07 | 1,08 | 2,58E-04 |
| rs1014971 | 22 | 39332623 | *APOBEC3A* | T | C | 1,12 | 7,85E-11 | 0,07 | 1,07 | 2,40E-04 |

*Estimates were aligned to allele associated with higher risk of bladder cancer, and the same allele in the previous and present GWAS.

†Estimates are for rs140584594, the lead variant in the *GSTM1* locus in the present GWAS.

Abbreviations: Chr, chromosome; EA, effect allele (bladder cancer increasing allele); GWAS, genome-wide association study; NEA, non-effect allele; OR, odds ratio; SNP, single-nucleotide polymorphism.
